# Lipid modulation of skeletal muscle mass and function

**DOI:** 10.1002/jcsm.12144

**Published:** 2016-10-08

**Authors:** Christopher Lipina, Harinder S Hundal

**Affiliations:** ^1^ Division of Cell Signalling and Immunology, Sir James Black Centre, School of Life SciencesUniversity of DundeeDundeeDD1 5EHUK

**Keywords:** Atrophy, Catabolism, Fatty acid, Lipid, mTOR, Obesity, Skeletal muscle

## Abstract

Loss of skeletal muscle mass is a characteristic feature of various pathologies including cancer, diabetes, and obesity, as well as being a general feature of ageing. However, the processes underlying its pathogenesis are not fully understood and may involve multiple factors. Importantly, there is growing evidence which supports a role for fatty acids and their derived lipid intermediates in the regulation of skeletal muscle mass and function. In this review, we discuss evidence pertaining to those pathways which are involved in the reduction, increase and/or preservation of skeletal muscle mass by such lipids under various pathological conditions, and highlight studies investigating how these processes may be influenced by dietary supplementation as well as genetic and/or pharmacological intervention.

## Introduction

**Figure 1:**
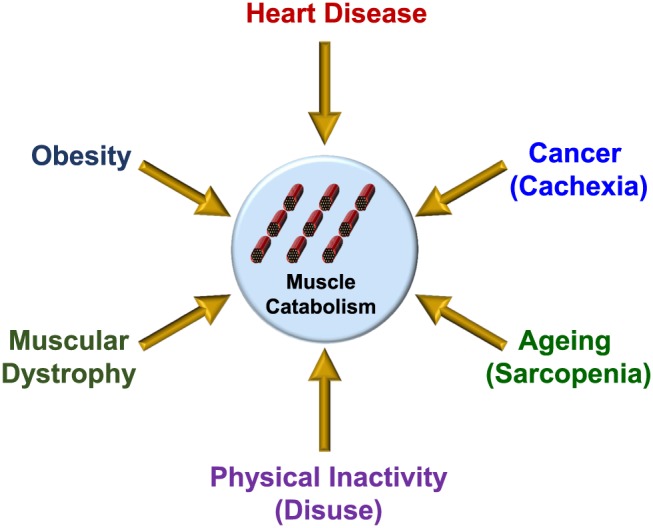
Disorders and conditions which are associated with reduced muscle mass and/or function. Schematic diagram illustrating various pathologies and/or conditions which are associated with increased muscle catabolism coinciding with reduced skeletal muscle mass and/or function.

The maintenance of skeletal muscle mass and integrity is crucial for proper functioning of the musculoskeletal system as well as efficient nutrient uptake and storage. Under normal physiological conditions, a network of interconnected signals serves to co‐ordinate muscle protein synthesis and proteolysis. However, any impairment of these signalling processes can contribute to a loss of muscle mass, or atrophy, which is a feature associated with various pathologies including cancer (termed cachexia), heart disease and obesity, as well as ageing (termed sarcopenia) (see *Figure* 1).^1^, ^2^, ^3^, ^4^, ^5^, ^6^, ^7^, ^8^, ^9^, ^10^, ^11^, ^12^ Moreover, injuries such as severe burns can induce a series of proinflammatory stress responses which have also been linked to muscle wasting post‐injury.^13^ Consequently, reduced skeletal muscle mass can severely weaken the musculoskeletal system and hamper locomotion, as well as contribute to the development of impaired glucose and lipid homeostasis, particularly in the obese state.

Conversely, muscle mass can be increased either through hypertrophy, which is characterized by an expansion in the size of pre‐existing myofibres, or through the process of hyperplasia, which involves an increase in the number of cells or fibres.^14^, ^15^, ^16^, ^17^ Indeed, several model systems have been used to study such growth responses in myogenic progenitor (satellite) cells and/or differentiated myotubes including, for example, the murine C2C12 muscle cell line (myoblasts originally cultured from thigh muscle of C3H mice), rat L6 muscle cells (a skeletal muscle cell line established from thigh muscle of newborn rats), or primary myogenic cells derived from purified muscle fibres.^18^, ^19^ Notably, muscle hypertrophy can be induced by multiple anabolic stimuli—among the most studied of which include insulin and insulin‐like growth factor 1 (IGF‐1).^20^ Indeed, signalling triggered by growth factors such as IGF‐1 act to positively regulate muscle growth, driven at least in part through the induction of protein synthesis.^21^, ^22^, ^23^ Mechanistically, activation of the IGF‐1/IGF‐1 receptor signalling axis leads to the insulin receptor substrate 1 (IRS1)‐dependent recruitment of PI3‐kinase and subsequent activation of protein kinase B (PKB) (also known as Akt) through the generation of phosphatidylintosiol‐3,4,5 trisphosphate (PIP3) by PI3‐kinase. Active Akt then promotes the activation of mechanistic target of rapamycin (mTOR) complex 1 (mTORC1) by phosphorylating and inhibiting its upstream repressor TSC2.^24^ This then results in the mTORC1‐dependent phosphorylation of p70‐S6 kinase 1 (S6K) and eIF4E‐binding protein (4E‐BP), leading to increased protein synthesis. An alternative downstream target of Akt is glycogen synthase kinase 3β (GSK3β) which becomes phosphorylated and inhibited by active Akt. Repression of GSK3 acts to relieve its inhibition of the initiation factor eIF2B, leading to increased protein synthesis.^25^ In addition, Akt also phosphorylates and inhibits the Forkhead box O (FOXO) family of transcription factors, thereby repressing their transcriptional activation of the E3 ubiquitin ligases Muscle Atrophy Fbox (MAFbx) (also known as atrogin‐1) and Muscle Ring Finger 1 (MuRF1) which function to promote ubiquitination and subsequent proteasomal degradation of target substrates.^9^ Pro‐inflammatory cytokines such as tumour necrosis factor alpha (TNF‐α) can also act to induce atrophic genes such as MuRF1 and atrogin‐1/MAFbx by activating the nuclear factor‐kappa B (NF‐kB) family of transcription factors.^26^ Therefore, a number of distinct signalling pathways have been implicated in controlling skeletal muscle hypertrophy and atrophy. Notably, diet‐induced obesity or short‐term high fat feeding has been shown to promote or augment muscle atrophy and catabolism, as evidenced by reduced muscle mass and muscle fibre size, in association with up‐regulated expression of atrophic factors (i.e. atrogin‐1/MAFbx and MuRF1) and increased rate of proteolysis.^10^, ^27^ Allied to this, fat infiltration into muscle has been associated with reduced muscle strength.^28^ Herein, we discuss the evidence which supports a role for the involvement of fatty acids and derived lipid metabolites in the regulation of skeletal muscle mass and function through their ability to modulate muscle cell growth, proliferation, and/or differentiation. Furthermore, we explore potential mechanisms that may be involved in the control of muscle hypertrophy/atrophy by such lipids.

## Fatty acid modulation of skeletal muscle mass and function

Evidence from several studies suggests that saturated and unsaturated fatty acids may act to differentially regulate skeletal muscle mass and function. For example, exposure of C2C12 myotubes to palmitate (C16:0), the most abundant circulating saturated fatty acid, has been shown to decrease myotube diameter and suppress insulin signalling.^29^ In accord with this, palmitate provision in muscle cells has been reported to induce the expression of pro‐atrophic genes such as atrogin‐1/MAFbx, concomitant with increased nuclear localization of its transcriptional regulator FoxO3.^30^ In contrast, application of docosahexaenoic acid (DHA), an omega‐3 polyunsaturated fatty acid (PUFA), did not alter myotube morphology when applied alone and was shown to counter‐modulate palmitate‐induced atrophy in C2C12 myotubes.^29^ Consistent with this, a separate study reported the amelioration of palmitate induced protein degradation in C2C12 myotubes following co‐treatment with DHA.^30^ Notably, this coincided with the ability of DHA to mitigate enhanced nuclear FoxO3 localization and atrogin‐1/MAFbx gene expression in response to palmitate provision.^30^

In accord with these findings in cultured muscle cells, several *in vivo* studies have also reported the ability of unsaturated fatty acids to convey beneficial responses which act to prevent muscle wasting and/or atrophy. For example, feeding mice bearing the colon‐26 adenocarcinoma, an animal model of cancer cachexia, with a diet supplemented with conjugated linoleic acid, was shown to preserve gastrocnemius muscle mass.^7^ Notably, this protective effect coincided with a reduction in skeletal muscle TNF‐α receptor expression suggesting that the PUFA may act to prevent muscle wasting, at least in part, by reducing the catabolic actions of the cytokine TNF‐α.^7^, ^31^ In a separate study, dietary supplementation with eicosapentaenoic acid (EPA; C20:5(n‐3)) attenuated protein degradation in gastrocnemius muscle of mice bearing the cachexia‐inducing MAC16 tumour.^4^ EPA treatment has also been reported to prevent arthritis‐induced reductions in gastrocnemius muscle weight in rats following administration of Freund's adjuvant, concomitant with the normalization of atrogin‐1/MAFbx and MuRF1 gene expression.^32^ Moreover, dystrophic hamsters fed a diet enriched in the PUFA α‐linolenic acid (ALA) (C18:3(n‐6)) exhibited improvements in muscle morphology and function, including enlarged myofibres.^33^ In accord with these findings, omega‐3 and omega‐6 PUFAs have also been shown to increase phosphorylation of p70S6K1 at Thr389, indicative of its increased activity, during myogenic differentiation of L6 myocytes.^34^ Together, these studies support the notion that unsaturated fatty acids can provide protection against muscle wasting in response to various pathological conditions. Furthermore, these findings highlight the distinct responses that saturated and unsaturated fatty acids induce to promote or counter muscle atrophy and protein degradation, respectively.

## Potential factors underlying fatty acid regulation of skeletal muscle size and mass

**Figure 2:**
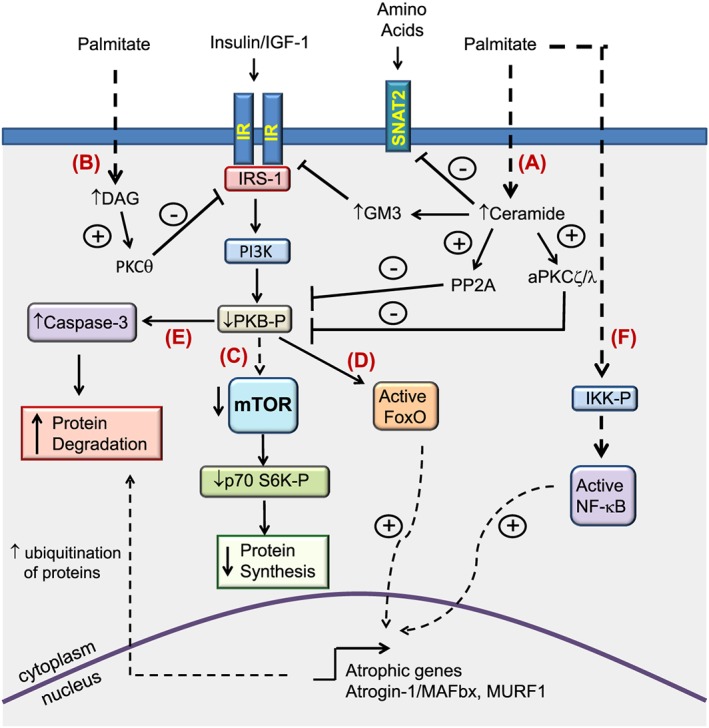
Summary of pathways mediating muscle atrophy by saturated fatty acids. Exposure of muscle cells to saturated fatty acids such as palmitate (C16:0) results in the intracellular accumulation of toxic lipid intermediates such as ceramide and diacylglycerol. (A) Increased ceramide levels can lead to the inhibition of protein kinase B/Akt through activation of atypical protein kinase C(ξ/λ) isoforms and/or protein phosphatase 2A. Moreover, ceramide acts as a precursor for the synthesis of the glycosphingolipid GM3 which has been shown to impair insulin receptor function. In addition, ceramide may also act to modulate nutrient uptake, for example by repressing the expression of the neutral amino transporter SNAT2 thereby reducing cellular amino acid supply. (B) Diacylglycerol‐induced stimulation of protein kinase Cθ has been shown to promote serine phosphorylation of IRS‐1, resulting in its impaired function. The resulting inhibition of protein kinase B/Akt in turn can lead to the repression of protein synthesis through suppression of mechanistic target of rapamycin (mTOR)/p70‐S6 kinase 1‐dependent signalling (C), the activation of Forkhead box O (FoxO) transcription factors and induction of their target atrophic genes (D), and/or the activation of caspase‐dependent proteolysis (E). In addition, stimulation of pro‐inflammatory signalling by long chain saturated fatty acids can lead to the nuclear factor‐kappa B‐dependent upregulation of atrophic genes (F).

A number of different signalling pathways and/or intermediates have been implicated as potential mediators of muscle wasting and atrophy, which themselves can be regulated in response to fatty acid provision (see *Figure* 2). For example, palmitate is known to act as a potent repressor of PKB/Akt directed signalling in skeletal muscle, at least in part through its ability to induce the accumulation of toxic lipid intermediates such as ceramide.^35^, ^36^ Indeed, such sphingolipids can act by stimulating protein phosphatase 2A (PP2A) or atypical protein kinase C (PKC) (PKCζ) isoforms to inhibit PKB/Akt.^37^ In accord with this, C2C12 myotube atrophy induced by TNF‐α has been reported to coincide with elevated levels of intracellular ceramide,^38^ whereas blocking ceramide synthesis has been shown to attenuate TNF‐α induced muscle atrophy in L6 myotubes, as well as protecting mice against tumour induced (*via* C26 carcinoma implantation) skeletal muscle atrophy *in vivo.* 38 Notably, these beneficial responses concurred with increased protein synthesis and decreased proteolysis, concomitant with reduced expression of the atrogin‐1/MAFbx gene *via* suppressed Foxo3 function, as well as increased abundance of key mediators of protein synthesis including S6K1 and PKB/Akt.^38^ Moreover, exogenous provision of ceramide in L6 muscle cells has been reported to reduce protein levels of the myogenic transcription factor myogenin *via* inhibition of phospholipase D, whilst inhibition of ceramide synthesis enhanced myogenin expression and accelerated myotube formation.^39^ A study by Turpin and colleagues also demonstrated increased muscle ceramide content following acute (5 h) intralipid® infusion, which coincided with the activation of pro‐apoptotic signalling as demonstrated by increased caspase‐3 activity in gastrocnemius muscle.^40^ However, the role of ceramide in promoting this lipid‐driven increase in muscle apoptosis was not investigated, for example by co‐administration of inhibitors of ceramide synthesis. Alternatively, elevated levels of ceramide associated with hyperlipidaemia may also act to suppress protein synthesis by inducing the expression and/or activity of key repressors of mTORC1‐S6K signalling such as Regulated in Development and DNA Damage 1 (REDD1).^41^, ^42^ Notably, it should also be highlighted that the ganglioside GM3 (trisialotetrahexosylganglioside), a sialic acid‐containing glycosphingolipid derived from ceramide, has also been implicated as a negative regulator of skeletal muscle growth and/or differentiation, concomitant with its reported ability to impair insulin action by impairing insulin receptor function.^43^, ^44^, ^45^, ^46^ Moreover, another ceramide derived lipid, ceramide‐1‐phosphate, has also been shown to stimulate C2C12 myoblast proliferation through a mechanism involving the activation of Akt, mTOR, and ERK1/2.^47^ Indeed, further work utilizing mice deficient for GM3 synthase, the enzyme responsible for the synthesizing GM3, may shed more light regarding the role of this ganglioside in the control of skeletal muscle mass, for example in response to obesity and/or aging.

In addition to sphingolipids, diacylglycerols (DAGs) are an alternative class of lipid which can be generated in response to fatty acid provision. Notably, increased levels of DAG have been associated with the development of insulin resistance.^35^, ^48^ Moreover, increased muscle DAG levels have been detected following lipid infusion in mice, concomitant with increased caspase‐3 activity in gastrocnemius muscle.^40^ Although little is known regarding the role of DAGs in the regulation of skeletal muscle mass, ex‐vivo mechanical activation of DAG kinaseζ (DGKζ), an enzyme which catalyzes the conversion of DAG to phosphatidic acid (PA), has been reported to promote increased mTOR dependent signalling and associated hypertrophy in isolated mouse extensor digitorum longus (EDL) muscle, concomitant with the reported ability of PA to bind and directly activate mTOR.^49^, ^50^ In accord with this, cardiac‐specific overexpression of DGKζ has also been shown to ameliorate myocardial atrophy in streptozotocin‐induced diabetic mice.^51^ Therefore, these findings suggest that activation and/or overexpression of DGKζ may provide a means of stimulating protein synthetic rates and hypertrophic responses, and thereby ameliorating losses in muscle mass, either through reducing cellular levels of DAG and/or increasing PA‐induced activation of mTOR signalling. Importantly, future work may involve investigating the potential beneficial effects of overexpressing of DGKζ in muscle as a means of countering age and/or diet induced muscle atrophy. In addition, animal models which exhibit elevated levels of DAG in skeletal muscle, including mice which are deficient for hormone‐sensitive lipase (HSL),^52^ may also be useful for elucidating the role of DAG in skeletal muscle atrophy.

Another important consideration relates to the possibility that distinct DAG species may impact differently on pathways that are involved in regulating muscle mass, for example as determined by the composition of the fatty acyl groups which become esterified at either the sn‐1,2, sn‐1,3, or the sn‐2,3 positions of the glycerol backbone of DAG.^53^, ^54^ Indeed, previous work by our group has demonstrated that treatment of rat L6 myotubes with palmitate leads to significant increases in the cellular levels of certain DAG species, as well as total cellular DAG content.^55^ Furthermore, co‐treatment with the monounsaturated fatty acid (MUFA) palmitoleate (C16:1) was shown to selectively suppress palmitate‐induced increases in the levels of DAG species containing C18:0 and C20:0 saturated fatty acyl moieties, coinciding with the MUFA's anti‐inflammatory action.^55^ Although not determined in this study, distinct stereoisomers of DAG may also differentially regulate muscle anabolic/catabolic signalling. To support this notion, sn‐1,2 DAG stereoisomers (in comparison to sn‐1,3 isomers) have been reported to be more potent at activating signalling pathways linked to insulin resistance, including the activation of PKC.^56^ Together, these studies provide emerging evidence that certain DAG molecules/isomers may play a more prominent role in the development of muscle atrophy, for example by promoting insulin resistance and/or increasing pro‐inflammatory drive. However, further work will be required to determine which of these DAG molecules, if any, are responsible for conveying muscle wasting actions. In an attempt to address this, future studies may involve treating cultured muscle cells with different DAG molecules/stereoisomers in order to determine their effects on myogenesis and/or muscle atrophy. Alternatively, further work may also incorporate detailed lipidomic analysis of various intramuscular DAG species in tissue isolated from animal models of muscle wasting, as well as monitoring potential changes in their abundance following interventions that are known to increase muscle mass (e.g. PUFA dietary provision or increased physical activity). Indeed, if such studies were to reveal a key role for DAG accumulation in the development of skeletal muscle atrophy, subsequent work may then involve determining the origin of such DAG species, for example by inhibiting the activity of enzymes implicated in DAG formation during triacylglycerol (TAG) synthesis (e.g. glycerol phosphate transferase (GPAT), acylglycerolphosphate acyltransferase (AGPAT), and lipin), or by altering the activity of enzymes implicated in TAG and/or DAG hydrolysis (e.g. adipose triglyceride lipase (ATGL) or HSL). To this end, previous work by Badin and co‐workers reported elevated ATGL protein abundance in skeletal muscle of type 2 diabetic individuals vs. lean control subjects, as well as reduced muscle HSL expression in obese individuals.^57^ In addition, the authors of the same study further demonstrated that overexpressing ATGL or inhibiting HSL activity in human primary myotubes resulted in the accumulation of cellular DAG and an associated impairment in insulin signalling. However, whether these changes in DAG levels are linked to muscle atrophy was not determined in this study.

As well as modulating PKB/Akt and/or mTORC1 directed signalling, fatty acids and/or their derived lipids may further contribute to muscle wasting by modulating nutrient (amino acid) transport and/or associated signalling. For example, previous work by our own group and others has demonstrated the ability of ceramide to down‐regulate the expression and/or activity of key nutrient transporters, including the neutral amino acid transporter SNAT2 (SLC38A2).^58^, ^59^ By doing so, fatty acids acting through such lipid intermediates may act to impair amino acid uptake, thereby contributing to a loss in muscle mass. Interestingly, in a separate study by our group, incubation of rat L6 myotubes with linoleic acid (C18:2) was shown to restrain adaptive upregulation of SNAT2 expression and activity in response to amino acid starvation.^60^ Notably, this fatty acid induced reduction in System A transport activity was mediated through increased ubiquitination and proteasomal degradation of SNAT2 protein.^60^ Conversely, in a separate study by Li and co‐workers, the mRNA expression of the amino acid transceptors LAT1 (an L‐type amino acid transporter) and SNAT2 were reported to be up‐regulated in the longissimus dorsi of pigs fed dietary n‐6 and n‐3 PUFAs.^61^ Hence, it is possible that fatty acids and/or their derived lipids may function to modulate adaptive strategies which are used by tissues such as skeletal muscle, in order to maximize or minimize nutrient uptake during conditions of fasting or nutrient deprivation.

## Role of unsaturated fatty acids in maintaining skeletal muscle size and mass

Importantly, current literature describes evidence to suggest that unsaturated fatty acids may act to counter pro‐atrophic mediators, including those triggered following exposure to saturated fatty acids. For example, MUFAs and PUFAs have been reported to prevent palmitate‐induced reductions in insulin sensitivity as well as conveying anti‐inflammatory effects in skeletal muscle cells.^55^, ^62^, ^63^ Indeed, NF‐kB dependent transcriptional regulation has been implicated in promoting disuse muscle atrophy in rat soleus muscle by increasing FoxO mediated activation of the MuRF1 promoter.^64^ Moreover, a recent study demonstrated that diminished anabolic signalling in skeletal muscle of aged mice coincided with the accumulation of intramuscular ceramide and DAG, as well as increased TNF‐α mRNA abundance.^65^ Interestingly, feeding fish oil to weaning piglets, which resulted in the enrichment of EPA, DHA, and total omega‐3 PUFA content within gastrocnemius muscle, coincided with a reduction in muscle TNF‐α levels and reduced expression of Toll‐like receptor 4 (TLR4), a target receptor for saturated fatty acids which stimulates pro‐inflammatory signalling in response to its activation.^66^ Notably, TLR4 stimulation by its ligand lipopolysaccharide has been reported to induce muscle catabolism in C2C12 myotubes through activation of the ubiquitin–proteasome and autophagy–lysosome pathways.^67^ In addition, DHA treatment of human muscle cells co‐cultured with macrophages has been shown to attenuate macrophage‐induced protein content of Fn14, a positive modulator of MuRF‐1 expression.^68^, ^69^ Therefore, based on these findings, it is conceivable that the reported anti‐inflammatory actions of unsaturated fatty acids in skeletal muscle cells may contribute, at least in part, to their ability to preserve muscle mass and/or function.

Notably, these protective actions may be linked to improvements in mitochondrial function, the impairment of which has been suggested to contribute to diet and/or age‐induced muscle atrophy.^27^, ^70^, ^71^ For example, a recent study by Roseno and colleagues reported that a short‐term (3 week) high fat diet augmented denervation muscle atrophy in mice by inducing protein degradation in mitochondria‐rich soleus, but not in glycolytic EDL muscle.^27^ Notably, 14 day denervation induced a loss in mitochondrial protein content in the soleus but not the EDL, regardless of diet. Therefore, these findings suggest that denervation‐induced loss of mitochondria and high fat diet‐induced impairment of mitochondrial function may combine to promote skeletal muscle atrophy.^27^ In contrast, an independent study by Tardif and co‐workers demonstrated that aged rats fed an oleate‐enriched diet display marked improvements in insulin sensitivity as well as increased muscle protein synthesis, concomitant with increased expression of genes implicated in stimulating mitochondrial β‐oxidation including peroxisome proliferator‐activated receptor (PPAR)α and PPARβ, as well as CPT‐1β.^72^, ^73^ Moreover, C2C12 myotubes treated with the PUFAs linolenic acid and ALA have been shown to exhibit increased activation of AMPK, another key positive regulator of mitochondrial β‐oxidation.^74^ In addition, DHA has also been reported to inhibit protein degradation in C2C12 myotubes through a PPARγ‐dependent pathway.^75^ Indeed, enhanced and/or preserved mitochondrial oxidative capacity, as previously reported in response to sole or co‐provision of unsaturated fatty acids, may also help prevent the intramuscular accumulation of lipotoxic intermediates such as ceramide which have been implicated in promoting muscle atrophy.^36^, ^55^, ^76^, ^77^ Furthermore, it is possible that PUFA supplementation may act to alter muscle contractile and metabolic properties, for example by promoting a shift from fast glycolytic to slow (oxidative) fibre types. To support this idea, previous work has demonstrated that feeding Wistar rats a diet enriched in n‐3 PUFAs results in the upregulation of proteins implicated in the activation of oxidative metabolism (e.g. mitochondrial uncoupling protein 3 and PPARγ coactivator 1‐α (PGC1α)) in EDL muscle (a fast‐type dominant muscle tissue).^78^ Interestingly, this PUFA‐mediated metabolic shift also coincided with reduced protein levels of the fast‐type MyHC‐2b (myosin heavy chain 2b) isoform in EDL muscle. Therefore, it is conceivable that a PUFA‐mediated shift towards a slow oxidative muscle fibre type may contribute, at least in part, towards beneficial gains in muscle mass and/or metabolic function.

Alternatively, regulation of muscle mass by lipids may also involve modulation of autophagy, a homeostatic mechanism which facilitates the degradation and recycling of proteins and organelles through the lysosomal machinery.^79^ Notably, increased autophagic degradation has been reported to coincide with muscle atrophy in various conditions and/or pathologies including cancer,^80^ denervation,^81^ as well as ageing.^80^, ^82^ Moreover, short‐term (3 week) high fat feeding has been shown to increase the abundance of autophagosome markers in denervated soleus of mice.^27^ In accord with this, Yuzefovych and co‐workers demonstrated increased autophagy in L6 myotubes following palmitate provision.^36^ Therefore, although a direct link has yet to be established *in vivo*, it is conceivable that altered protein turnover *via* autophagy may, at least in part, mediate lipid‐induced alterations in muscle mass.

It should also be highlighted that certain unsaturated fatty acids can alter the proliferation rate of satellite cells which function as myogenic progenitor cells required for muscle growth and regeneration. For example, DHA and EPA have been shown to inhibit proliferation of C2C12 myoblasts as well as satellite cells isolated from turkey muscle.^83^, ^84^ Notably, these growth suppressing actions have been linked to reduced levels of cyclin E and CDK2, proteins which play a critical role in cell cycle progression, as well as suppressed activation of ERK1/2, a mitogen‐activated protein kinase implicated in promoting cell growth and division.^84^, ^85^ In contrast, feeding dystrophic δ‐sarcoglycan deficient hamsters a diet enriched in ALA (an omega‐3 PUFA) was demonstrated to increase satellite cell proliferation and differentiation in EDL muscle, concomitant with improved muscular histology.^33^ Notably, these beneficial responses coincided with the ability of ALA to increase the proportion of α‐MHC positive myofibres in skeletal muscle of dystrophic hamsters, along with a reduction in β‐MHC expression, thereby contributing to the preservation of a more physiological α/β MHC ratio. Moreover, in the same study, supplementation of dietary ALA was also shown to prevent the aberrant cytoplasmic accumulation of key membrane proteins in the adductor muscles of dystrophic hamsters including caveolin‐3, a protein involved in regulating cell adhesion and membrane repair, as well as being implicated in the control of muscle differentiation and insulin induced signalling.^33^, ^86^, ^87^ Indeed, given the fact that aberrations in caveolin‐3 function and/or localization have been associated with various skeletal muscle disease phenotypes,^88^, ^89^, ^90^, ^91^, ^92^ it is plausible that fatty acids and/or their lipid derivatives may influence satellite cell proliferation and/or muscle differentiation, at least in part, by altering the function and/or subcellular localization of caveolin isoforms, as well as other key structural membrane components.

The effects of fatty acids upon muscle mass and differentiation may also be mediated through a number of derived lipid metabolites. For example, the ability of the PUFA arachidonic acid (C20; 4n‐6) to increase the size, myonuclear content and protein content of C2C12 myotubes has been shown to be mediated through cyclooxygenase‐2 (COX‐2) activity, implying dependency on downstream prostaglandin synthesis.^93^ In accord with this, arachidonic acid induced growth of C2C12 myocytes was reported to coincide with increased secretion of the eicosanoids PGF(2α) and PGE.^2^, ^93^ It is noteworthy that several studies have also documented the positive role that prostaglandins play in promoting early cell surface events, including cell–cell adhesion, which subsequently mediate the fusion of myoblasts into myotubes.^94^, ^95^ Indeed, important follow‐up studies may involve determining the exact identity of the molecular targets through which prostaglandins mediate their actions, for example by acting upon G‐protein linked prostanoid receptors (e.g. EP1).^94^, ^96^ In contrast, another arachidonic acid derived lipid metabolite known as 2‐arachidonoylglycerol (2‐AG), a key endogenous lipid ligand of the endocannabinoid system, has recently been reported to inhibit differentiation of primary human satellite cells and murine C2C12 myoblasts by targeting the G‐protein coupled cannabinoid receptor 1, and its subsequent inhibition of Kv7.4 channels.^97^ Therefore, the possible involvement of such lipid intermediates in regulating muscle catabolism in response to certain pathological conditions cannot be excluded.

## Lipid modulation of muscle mass and function: a human perspective

It is well established that there is a progressive loss in skeletal muscle mass and its regenerative capacity in response to aging in humans.^9^, ^98^, ^99^ Moreover, increased adiposity as observed in aging has been linked to altered muscle protein synthetic responses in aged individuals.^100^ Therefore, it is conceivable that changes in lipid levels and/or composition may contribute to muscle atrophy in these conditions. To support this idea, there is evidence to suggest that modifying dietary composition may impact upon muscle mass and/or function in humans. For example, a study by McGlory and co‐workers reported that consumption of fish oil increased the omega‐3 PUFA content of muscle (*Vastus lateralis*) in healthy male individuals, which coincided with elevated expression of anabolic signalling proteins including mTOR.^101^ Moreover, inclusion of dietary MUFAs and PUFAs has been reported to reduce the expression of lipogenic genes in skeletal muscle of insulin resistant subjects, concomitant with reduced fractional synthetic rates of intramuscular DAG and triacylglycerols.^102^ Notably, these beneficial responses may be linked to improvements in insulin sensitivity conveyed by dietary supplementation of omega‐3 PUFAs in humans.^103^, ^104^, ^105^

As well as dietary interventions, resistance training has also been reported to enhance skeletal muscle innervation in obese older adults, as well as downregulating atrophic markers in skeletal muscle of mice in various models of atrophy.^106^, ^107^ Moreover, a study by Mikkelsen and colleagues demonstrated significantly increased thigh muscle area in aged individuals which had undergone life‐long endurance (running) exercise, compared with age‐matched untrained counterparts.^108^ In accord with these findings, physical exercise training was shown to normalize the levels of atrophic modulators TNF‐α, Murf‐1 and atrogin‐1/MAFbx in the myocardium following the induction of heart failure in rats.^109^ Therefore, increased physical activity may be used as an alternative and/or additional strategy to counteract the deleterious effects of aging and/or obesity upon muscle mass loss, potentially through countering lipid‐induced insulin resistance and/or chronic low grade inflammation in skeletal muscle.^108^, ^110^, ^111^, ^112^, ^113^, ^114^ Intriguingly, it has been previously reported that dietary supplementation with unsaturated fatty acids may also act to improve physical performance and/or enhance the beneficial metabolic effects associated with exercise, particularly in sedentary or untrained individuals.^115^ To support this idea, low dietary intake of tuna fish oil has been shown to promote resistance to muscle fatigue in rats, concomitant with a selective increase in DHA membrane phospholipid content within gastrocnemius muscle.^116^, ^117^ Therefore, a more integrated approach involving modifications to dietary fat consumption as well as increased physical exercise may provide a more effective strategy to alleviate the deleterious effects associated with muscle atrophy.

## Conclusions and future perspectives

**Figure 3:**
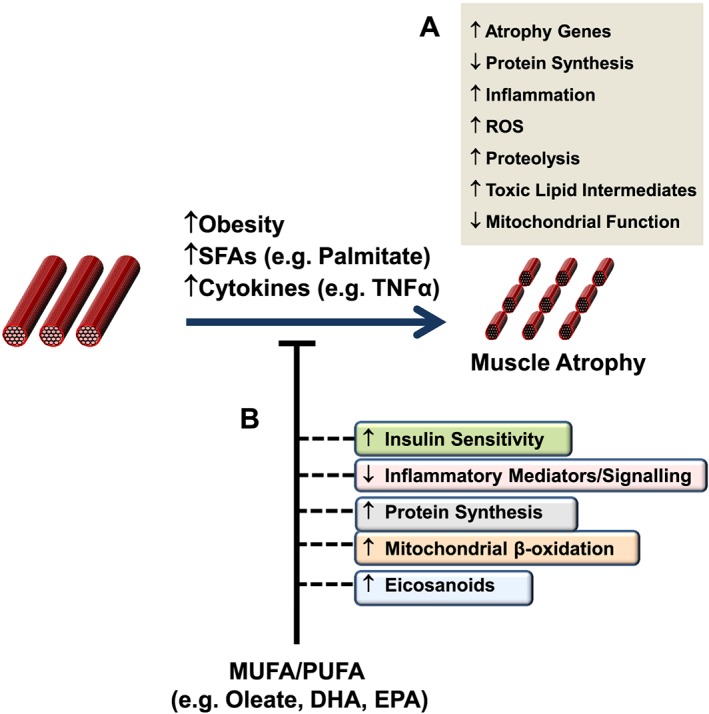
Potential mechanisms by which unsaturated fatty acids may counter obesity and/or fatty acid induced skeletal muscle atrophy. Elevated levels of saturated fatty acids and/or pro‐inflammatory cytokines, for example during obesity, can promote the development of skeletal muscle atrophy through various pathways and processes as indicated (A). Importantly, monounsaturated fatty acids and polyunsaturated fatty acids have been shown to counter these pro‐atrophic actions, which may be mediated through their ability to increase insulin sensitivity and the production of protective eicosanoids, as well as enhancing mitochondrial oxidative capacity and protein synthesis, whilst concomitantly reducing pro‐inflammatory drive (B).

To conclude, there is growing appreciation that fatty acids and/or their lipid derivatives can play an important role in modulating skeletal muscle mass and function. Collectively, the evidence presented in this review indicates that saturated fatty acids act to convey detrimental effects upon muscle function, for example by impairing or inducing protein synthesis and catabolism, respectively (see *Figure* 2). In contrast, a number of different unsaturated fatty acids have been shown to counteract many of the pro‐catabolic actions associated with saturated fatty acid provision (*Figure* 3). However, further work will be required to delineate the pathways and processes underlying fatty acid‐induced muscle atrophy, as well as those mediating improvements in muscle function in response to the provision of unsaturated fatty acids (i.e. increased protein synthesis, reduced atrophy, improved metabolic function) (*Figure* 3). To this end, strategies aimed at altering intramuscular lipid content and/or composition under those conditions which can promote muscle wasting (e.g. increased obesity, ageing, physical inactivity), for example by suppressing the accumulation of lipid mediators such as ceramides (e.g. through inhibiting *de novo* ceramide synthesis) or prostaglandins (e.g. by inhibiting COX‐2 activity), may provide useful insight into the role that distinct classes of lipids play in the modulation of muscle mass and function. Importantly, such work is likely to involve the use of relevant animal models or human subjects that would require to take into consideration factors such as genetic background as well as dietary composition and caloric intake. In addition, these studies may also involve determining potential lipid induced alterations to muscle architecture and fibre type composition which can influence muscle strength, as well as monitoring changes in intramuscular signalling and metabolites within specific muscle fibre types. Allied to this, further work exploring the role of fatty acids and lipid intermediates in regulating the proliferation, differentiation and/or function of human muscle derived satellite cells and primary myotubes would need to be performed in order to make appropriate comparisons with data obtained in other experimental models (e.g. C2C12 myotubes), which have been shown to exhibit functional differences (e.g. in their level of maturation).^118^ Collectively, data obtained from such studies may lead to the development of novel therapeutic strategies to counteract muscle atrophy, and/or improve regenerative capacity following injury or disease.

## Conflict of interest

The authors declare that there are no conflicts of interest that require to be declared.
